# Association of Post–COVID-19 Condition Symptoms and Employment Status

**DOI:** 10.1001/jamanetworkopen.2022.56152

**Published:** 2023-02-15

**Authors:** Roy H. Perlis, Kristin Lunz Trujillo, Alauna Safarpour, Mauricio Santillana, Katherine Ognyanova, James Druckman, David Lazer

**Affiliations:** 1 Massachusetts General Hospital, Boston; 2 Department of Psychiatry, Harvard Medical School, Boston, Massachusetts; 3 Northeastern University, Boston, Massachusetts; 4 Harvard University, Cambridge, Massachusetts; 5 Rutgers University, New Brunswick, New Jersey; 6 Northwestern University, Evanston, Illinois

## Abstract

**Importance:**

Little is known about the functional correlates of post–COVID-19 condition (PCC), also known as long COVID, particularly the relevance of neurocognitive symptoms.

**Objective:**

To characterize prevalence of unemployment among individuals who did, or did not, develop PCC after acute infection.

**Design, Setting, and Participants:**

This survey study used data from 8 waves of a 50-state US nonprobability internet population-based survey of respondents aged 18 to 69 years conducted between February 2021 and July 2022.

**Main Outcomes and Measures:**

The primary outcomes were self-reported current employment status and the presence of PCC, defined as report of continued symptoms at least 2 months beyond initial month of symptoms confirmed by a positive COVID-19 test.

**Results:**

The cohort included 15 308 survey respondents with test-confirmed COVID-19 at least 2 months prior, of whom 2236 (14.6%) reported PCC symptoms, including 1027 of 2236 (45.9%) reporting either brain fog or impaired memory. The mean (SD) age was 38.8 (13.5) years; 9679 respondents (63.2%) identified as women and 10 720 (70.0%) were White. Overall, 1418 of 15 308 respondents (9.3%) reported being unemployed, including 276 of 2236 (12.3%) of those with PCC and 1142 of 13 071 (8.7%) of those without PCC; 8229 respondents (53.8%) worked full-time, including 1017 (45.5%) of those with PCC and 7212 (55.2%) without PCC. In survey-weighted regression models excluding retired respondents, the presence of PCC was associated with a lower likelihood of working full-time (odds ratio [OR], 0.71 [95% CI, 0.63-0.80]; adjusted OR, 0.84 [95% CI, 0.74-0.96]) and with a higher likelihood of being unemployed (OR, 1.45 [95% CI, 1.22-1.73]; adjusted OR, 1.23 [95% CI, 1.02-1.48]). The presence of any cognitive symptom was associated with lower likelihood of working full time (OR, 0.70 [95% CI, 0.56-0.88]; adjusted OR, 0.75 [95% CI, 0.59-0.84]).

**Conclusions and Relevance:**

PCC was associated with a greater likelihood of unemployment and lesser likelihood of working full time in adjusted models. The presence of cognitive symptoms was associated with diminished likelihood of working full time. These results underscore the importance of developing strategies to treat and manage PCC symptoms.

## Key Points

**Question:**

Is post–COVID-19 condition (PCC), also known as long COVID, associated with differences in employment status that might suggest functional impairment?

**Findings:**

Among 15 308 individuals with prior COVID-19 infection, those with PCC were less likely to be employed full-time and more likely to be unemployed. These differences persisted after adjustment for demographic differences between those with and without PCC.

**Meaning:**

These findings suggest that individuals with PCC are less likely to be working and to be working full time.

## Teaser

This survey study evaluates the association of post–COVID-19 condition symptoms with employment status.

## Introduction

Symptoms of COVID-19 infection persist beyond 2 months in a subset of individuals, a phenomenon referred to as post–COVID-19 condition (PCC), also known as long COVID.^1^ This syndrome has become prevalent,^2,3,4^ but little is known about its association with functioning, with a 15-month follow-up study^5^ describing an association with poorer quality of life and diminished function.

A further question is whether particular illness features, such as neurocognitive symptoms, are associated with differential functioning, with a prior small study^6^ associating these symptoms with decreased quality of life. A range of studies suggest that central nervous system changes may persist following acute infection,^7^ and cognitive symptoms are common in PCC.^2^ We therefore examined whether PCC, and neurocognitive symptoms in particular, are associated with differential rates of employment as a proxy for functional impairment, to better guide development of potential interventions.

## Methods

In this survey study, we analyzed a previously described cohort derived from 8 waves of a nonprobability-sample internet survey called the COVID States Project, conducted every 4 to 8 weeks between February 2021 and July 2022.^2^ Respondents were US residents aged 18 years and older who provided written informed consent electronically before completing the survey online, according to a protocol approved by the institutional review board of Harvard University. We report results in accordance with American Association for Public Opinion Research (AAPOR) reporting guidelines.

The cohort included all individuals younger than 70 years who reported a positive COVID-19 test result, either polymerase chain reaction or antigen-based, at least 2 months before the survey month. Respondents were asked whether their acute symptoms had resolved; those who reported that they had not were then asked to complete a checklist of commonly reported symptoms. From this checklist of 25 items,^2^ we combined 2 (memory problems and difficulty concentrating or focusing, or brain fog) for further evaluation. Respondents also self-reported sociodemographic variables, including race and ethnicity selected from a list according to US Census categories (African American, Asian, Hispanic, White, and other [Native American, Pacific Islander, other]), to allow for survey weighting to match the US adult population. For purposes of analysis and reporting, because they represented fewer than 5% of the total cohort, Native American, Pacific Islander, and other were combined into a single group.

### Statistical Analysis

Primary analyses examined associations between PCC and lack of full employment or unemployment among those who were not retired, adjusted for age, sex, race and ethnicity, education, urbanicity (urban, suburban, or rural), and region, using multiple logistic regression with interlocking survey weights in R statistical software version 4.0 (R Project for Statistical Computing). All *P* values are 2-sided, with significance considered to be an uncorrected value of *P *< .05.

## Results

The cohort included 15 308 survey respondents aged 18 to 69 years with test-confirmed COVID-19 at least 2 months before date of survey completion, of whom 2236 (14.6%) reported PCC symptoms (Table). The mean (SD) age was 38.8 (13.5) years; 9679 participants (63.2%) identified as women and 10 720 (70.0%) were White.

**Table.** Features of Survey Respondents With Prior COVID-19 Illness Who Did or Did Not Report Current PCC Symptoms

Characteristic	Respondents, No. (%)			*P* value
	Recovered (n = 13 072)	PCC (n = 2236)	Total (N = 15 308)	
Sex				
Male	5115 (39.1)	514 (23.0)	5629 (36.8)	<.001
Female	7957 (60.9)	1722 (77.0)	9679 (63.2)	
Age, mean (SD), y	38.2 (13.3)	42.0 (13.9)	38.8 (13.5)	<.001
Education				
High school or less	3176 (24.3)	600 (26.8)	3776 (24.7)	<.001
Some college	4119 (31.5)	923 (41.3)	5042 (32.9)	
Bachelor’s degree	3416 (26.1)	489 (21.9)	3905 (25.5)	
Graduate degree	2361 (18.1)	224 (10.0)	2585 (16.9)	
Urbanicity				
Rural	2049 (15.7)	446 (19.9)	2495 (16.3)	<.001
Suburban	7222 (55.3)	1351 (60.4)	8573 (56.0)	
Urban	3800 (29.1)	439 (19.6)	4239 (27.7)	
Race and ethnicity				
African American	1336 (10.2)	165 (7.4)	1501 (9.8)	<.001
Asian	745 (5.7)	65 (2.9)	810 (5.3)	
Hispanic	1584 (12.1)	230 (10.3)	1814 (11.9)	
Other race^a^	382 (2.9)	81 (3.6)	463 (3.0)	
White	9025 (69.0)	1695 (75.8)	10 720 (70.0)	
US region				
Northeast	2066 (15.8)	295 (13.2)	2361 (15.4)	<.001
Midwest	3258 (24.9)	622 (27.8)	3880 (25.3)	
South	5088 (38.9)	907 (40.6)	5995 (39.2)	
West	2660 (20.4)	412 (18.4)	3072 (20.1)	
Current employment				
Employed				<.001
Full time	7212 (55.2)	1017 (45.5)	8229 (53.8)	
Part time	1508 (11.5)	246 (11.0)	1754 (11.5)	
Self-employed	600 (4.6)	149 (6.7)	749 (4.9)	
Unemployed	1142 (8.7)	276 (12.3)	1418 (9.3)	
Homemaker	847 (6.5)	191 (8.5)	1038 (6.8)	
Student	797 (6.1)	98 (4.4)	895 (5.8)	
Retired	916 (7.0)	243 (10.9)	1159 (7.6)	
Gig or contract worker	50 (0.4)	16 (0.7)	66 (0.4)	
Any employment	9370 (71.7)	1428 (63.9)	10 798 (70.5)	<.001
Among currently unemployed^b^				
Employed full time prepandemic	306 (28.4)	103 (39.6)	409 (30.5)	<.001
Currently looking for work	717 (63.8)	157 (57.9)	874 (62.7)	.07

Abbreviation: PCC, post–COVID-19 condition.

^a^ Other race includes Native American, Pacific Islander, and “other” indicated on self-report race and ethnicity checklist.

^b^ Prepandemic employment status was available for only 1079 individuals without PCC and 260 with PCC; current job-seeking status was available for only 1124 individuals without PCC and 271 with PCC.

Overall, 1418 of 15 308 respondents (9.3%) reported being unemployed, including 276 of 2236 (12.3%) of those with PCC and 1142 of 13 072 (8.7%) of those without PCC; 8229 (53.8%) worked full time, including 1017 (45.5%) of those with PCC and 7212 (55.2%) without PCC. For the full cohort, 10 798 (70.5%) reported any employment, including 1428 (63.9%) with PCC and 9370 (71.7%) without PCC.

A subset of currently unemployed individuals were asked about employment status before the pandemic (Table). Unemployed individuals with PCC were more likely to have been employed full time before the pandemic than those without PCC (103 of 260 respondents [39.6%] vs 306 of 1079 respondents [28.4%]; χ^2^_1_ = 12.5; *P* < .001).

**Figure 1.**
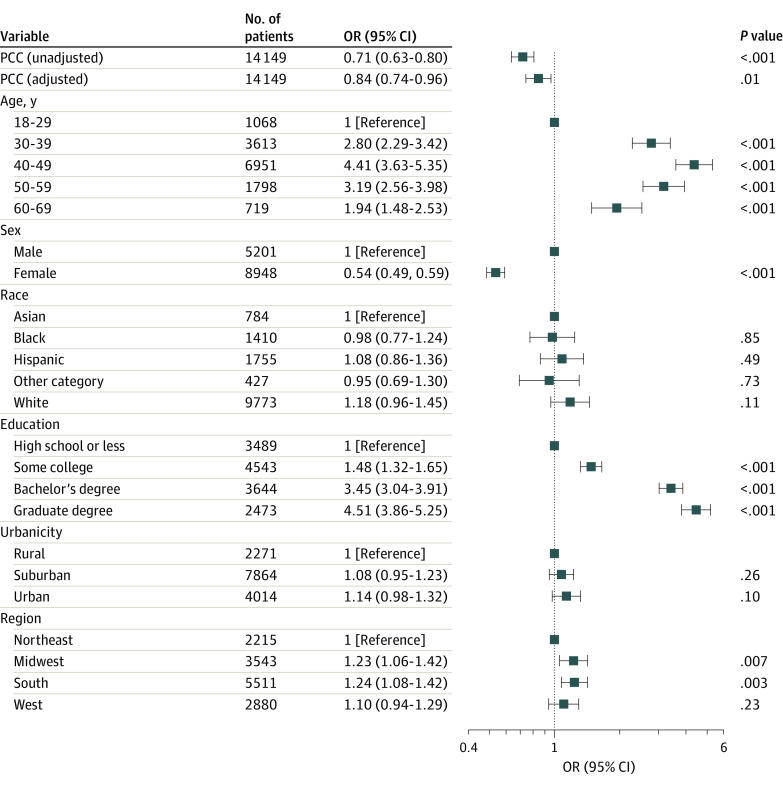
Association Between Post–COVID-19 Condition (PCC) and Likelihood of Working Full Time at Time of Survey in Logistic Regression Models Without and With Adjustment for Sociodemographic Features Other race includes Native American, Pacific Islander, and any other race. OR indicates odds ratio.

**Figure 2.**
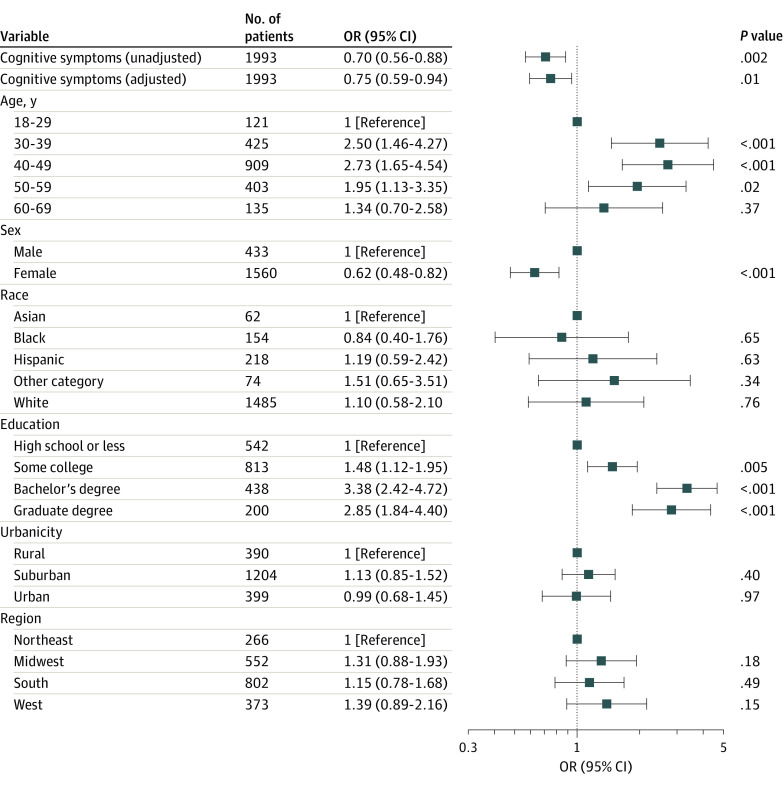
Among Individuals With Post–COVID-19 Condition, Association Between Neurocognitive Symptoms and Likelihood of Working Full Time at Time of Survey in Logistic Regression Models Without and With Adjustment for Sociodemographic Features Other race includes Native American, Pacific Islander, and any other race. OR indicates odds ratio.

In survey-weighted regression models excluding retired respondents, the presence of PCC was associated with lower likelihood of working full time (odds ratio [OR], 0.71 [95% CI, 0.63-0.80]; adjusted OR, 0.84 [95% CI, 0.74-0.96]) (Figure 1) and with a higher likelihood of being unemployed (OR, 1.45 [95% CI, 1.22-1.73]; adjusted OR, 1.23 [95% CI, 1.02-1.48]). We next examined whether specific symptoms of PCC were associated with likelihood of full-time employment. Among those with PCC, 1027 of 2236 respondents (45.9%) reported either brain fog (909 respondents [40.7%]) or impaired memory (634 respondents [28.4%]). The presence of any cognitive symptom was associated with lower likelihood of working full time (OR, 0.70 [95% CI, 0.56-0.88]; adjusted OR, 0.75 [95% CI, 0.59-0.84]) (Figure 2).

## Discussion

Among 15 308 US adults surveyed between February 2021 and March 2022, PCC was associated with a greater likelihood of unemployment and lesser likelihood of working full time in adjusted models. Furthermore, among those with PCC, the presence of cognitive symptoms was associated with diminished likelihood of working full time, extending recent reports associating cognitive symptoms with poor quality of life among employed individuals.^6^ As unemployed individuals with PCC were more likely to have been employed before the pandemic, it is unlikely that employment status simply reflects a factor associated with risk for reporting PCC (ie, reverse causation), or confounding by some characteristic that contributes to unemployment more generally.

### Limitations

A limitation in our analysis is the reliance on cross-sectional data, precluding determinations of causation. Moreover, the extent to which our results generalize remains to be determined, although prior work strongly suggests consistency with other approaches.^8^

## Conclusions

The results of this survey study underscore the importance of developing strategies to respond to PCC, and particularly the associated neurocognitive symptoms. Whether rehabilitation strategies drawn from neurology and psychiatry can help to ameliorate the impact of such symptoms merits investigation. More generally, although the true economic impact of the pandemic is difficult to estimate, these results underscore the importance of considering persistent effects of lost productivity.

## Notes

Supplement.
Data Sharing Statement
