# Supplementary material for: Association of Post–COVID-19 Condition Symptoms and Employment Status
Source: JAMA Netw Open. 2023 Feb 15;6(2):e2256152. doi: 10.1001/jamanetworkopen.2022.56152 (PMC9932847; doi:10.1001/jamanetworkopen.2022.56152)
Supplement: Supplement. — Data Sharing Statement [file jamanetwopen-e2256152-s001.pdf]

Data Sharing Statement

Perlis. Association of Post-COVID-19 Condition Symptoms and Employment Status. *JAMA Netw Open*. Published February 15, 2023. doi:10.1001/jamanetworkopen.2022.56152

Data
Data available: No
